# CUDASW++2.0: enhanced Smith-Waterman protein database search on CUDA-enabled GPUs based on SIMT and virtualized SIMD abstractions

**DOI:** 10.1186/1756-0500-3-93

**Published:** 2010-04-06

**Authors:** Yongchao Liu, Bertil Schmidt, Douglas L Maskell

**Affiliations:** 1 School of Computer Engineering, Nanyang Technological University, Singapore

## Abstract

**Background:**

Due to its high sensitivity, the Smith-Waterman algorithm is widely used for biological database searches. Unfortunately, the quadratic time complexity of this algorithm makes it highly time-consuming. The exponential growth of biological databases further deteriorates the situation. To accelerate this algorithm, many efforts have been made to develop techniques in high performance architectures, especially the recently emerging many-core architectures and their associated programming models.

**Findings:**

This paper describes the latest release of the CUDASW++ software, CUDASW++ 2.0, which makes new contributions to Smith-Waterman protein database searches using compute unified device architecture (CUDA). A parallel Smith-Waterman algorithm is proposed to further optimize the performance of CUDASW++ 1.0 based on the single instruction, multiple thread (SIMT) abstraction. For the first time, we have investigated a partitioned vectorized Smith-Waterman algorithm using CUDA based on the virtualized single instruction, multiple data (SIMD) abstraction. The optimized SIMT and the partitioned vectorized algorithms were benchmarked, and remarkably, have similar performance characteristics. CUDASW++ 2.0 achieves performance improvement over CUDASW++ 1.0 as much as 1.74 (1.72) times using the optimized SIMT algorithm and up to 1.77 (1.66) times using the partitioned vectorized algorithm, with a performance of up to 17 (30) billion cells update per second (GCUPS) on a single-GPU GeForce GTX 280 (dual-GPU GeForce GTX 295) graphics card.

**Conclusions:**

CUDASW++ 2.0 is publicly available open-source software, written in CUDA and C++ programming languages. It obtains significant performance improvement over CUDASW++ 1.0 using either the optimized SIMT algorithm or the partitioned vectorized algorithm for Smith-Waterman protein database searches by fully exploiting the compute capability of commonly used CUDA-enabled low-cost GPUs.

## Background

Sequence database searches in the field of bioinformatics are used to identify potential evolutionary relationships by means of identifying the similarities between query and subject sequences. The similarities between sequences can be determined by computing their optimal local alignments using the dynamic programming based on the Smith-Waterman (SW) algorithm [1,2]. However, the quadratic time complexity of this algorithm makes it computationally demanding, which is further compounded by the exponential growth of sequence databases. To reduce the execution time, some heuristic solutions, such as FASTA [3] and BLAST [4,5], have been devised to reduce the execution time, usually producing good results. However, these heuristics might fail to detect some distantly related sequences due to the loss of sensitivity. In this case, the use of high performance architectures, especially the emerging accelerator technologies and many-core architectures such as FPGAs, Cell/BEs and GPUs, becomes one recent trend to execute the SW algorithm, allowing the production of exact results in a reasonably shorter time.

For the FPGA technology, linear systolic array and massively parallel computing using custom instructions are used to perform the SW algorithm. Oliver et al. [6,7] constructed a linear systolic array to perform the SW algorithm on a standard Virtex II FPGA board using affine gap penalties. Li et al. [8] exploits custom instructions to accelerate the SW algorithm for DNA sequences on an Altera Stratix EP1S40 FPGA by dividing the SW matrix into grids of 8 × 8 cells. For the SIMD vectorization, particularly streaming SIMD extensions 2 (SSE2) technology, there are two basic vectorized SW algorithms available: one computes the algorithm using SIMD vectors parallel to the minor diagonal [9], and the other uses SIMD vectors parallel to the query sequence in a sequential layout [10] or a striped layout [11]. The striped SW approach [11] was then optimized for the Cell/BE [12]. SWPS3 [13] extends this work for the Cell/BE and also improves it for ×86/SSE2 to support multi-core processors, and CBESW [14] is designed for the Cell/BE-based PlayStation 3. For general-purpose GPUs, Liu et al. [15] developed an implementation of the SW algorithm using OpenGL as a first step. After the advent of CUDA programming model, SW-CUDA [16] was developed using CUDA, supporting multiple G80 (and higher) GPUs. However, this algorithm distributes the SW computation among multi-core CPUs and GPUs, which makes it highly CPU dependent and not able to truly exploit the computation power of GPUs. Different from SW-CUDA, CUDASW++ 1.0 [17], designed for multiple G200 (and higher) GPUs, completes all the SW computations on GPUs by fully exploiting the powerful GPUs. To the best of our knowledge, CUDASW++ 1.0 was the fastest publicly available solution to the exact SW algorithm on commodity hardware before this paper.

In this paper, we present the latest release of the CUDASW++ software, CUDASW++ 2.0, which makes new contributions to SW protein database searches using CUDA by deeply exploring the compute power of CUDA-enabled GPUs. An optimized SIMT SW algorithm is suggested to further optimize the performance of CUDASW++ 1.0 based on the SIMT abstraction. For the first time have we investigated a partitioned vectorized SW algorithm using CUDA based on the virtualized SIMD abstraction. CUDASW++ 2.0 obtains significant performance improvement over CUDASW++ 1.0 using either the optimized SIMT or the partitioned vectorized algorithms on the same platforms, achieving a performance of up to 17 (30) GCUPS on a single-GPU GeForce GTX 280 (dual-GPU GeForce GTX 295) graphics card. In addition, it also outperforms the other previous SW sequence database search implementations on GPUs and some other implementations using SSE2, Cell/B.E or heuristics.

### The Smith-Waterman algorithm

The SW algorithm is used to identify the similarity between two sequences by computing the maximum local alignment score. Given two sequences *S*_1 _and *S*_2 _of lengths *l*_1 _and *l*_2 _respectively, the SW algorithm computes the similarity score *H*(*i*, *j*) of two sequences ending at position *i *and *j *of *S*_1 _and *S*_2_, respectively. *H*(*i*, *j*) is computed as in equation (1), for 1 ≤ *i *≤ *l*_1_, 1 ≤ *j *≤ *l*_2_:(1)

where *sbt *is the substitution matrix, *ρ *is the gap open penalty and *σ *is the gap extension penalty. A substitution matrix *sbt *gives the substitution rates of amino acids in proteins, derived from alignments of protein sequences. The recurrences are initialized as *H*(*i*, 0) = *H*(0, *j*) = *E*(*i*, 0) = *F*(0, *j*) = 0 for 0 ≤ *i *≤ *l*_1 _and 0 ≤ *j *≤ *l*_2_. The maximum local alignment score is defined as the maximum score in *H*. The computation of each cell in *H *depends on its left, upper, and upper-left neighbors, as shown by the three arrows in Additional file 1. In addition, this data dependency implies that all cells on the same minor diagonal in the alignment matrix are independent, and can be computed in parallel. Thus, the alignment can be computed in minor-diagonal order from the top-left corner to the bottom-right corner in the alignment matrix, where the computation of minor diagonal *i *only needs the results of minor diagonals *i*-1 and *i*-2.

### CUDA programming model

CUDA is an extension of C/C++ with a minimalist set of abstractions for expressing parallelism, enabling users to write scalable multi-threaded parallel code for CUDA-enabled GPUs [18]. A CUDA program consist of two parts: a host program running on the host CPU, and one or more parallel *kernels *which can be executed on GPUs with NVIDIA's Tesla unified graphics and computing architecture [19].

A kernel is written in conventional scalar C-code, which represents the operations to be performed by a single thread and is invoked as a set of concurrently executing threads. These threads are organized into a grid of thread blocks, where a thread block is a set of concurrent threads. This hierarchical organization has implications for thread communication and synchronization. Threads in a thread block are allowed to synchronize with each other using barriers, and can communicate through a *per-block shared memory *(PBSM). However, threads located in different thread blocks cannot communicate or synchronize directly. To write efficient CUDA programs, besides the PBSM, it is important to understand the other memory spaces in more detail: non-cached global and local memory, cached texture and constant memory as well as on-chip registers.

The Tesla architecture is built around a fully programmable scalable processor array, organized into a number of streaming multiprocessors (SMs). Each SM contains eight scalar processors (SPs), sharing a PBSM of size 16 KB. All threads of a thread block are executed concurrently on a single SM. The SM executes threads in small groups of 32 threads, called *warps*, in an SIMT fashion. When one thread blocks is scheduled to execute on an SM, threads in the thread block are split into warps that get scheduled by the SIMT unit. A warp executes one common instruction at a time, but allows for instruction divergence. When divergence occurs, the warp serially executes each branch path. Thus, parallel performance is generally penalized by data-dependent conditional branches and improved if all threads in a warp follow the same execution path. Branch divergence occurs only in a warp, and different warps run independently regardless of common or disjointed code paths they are executing.

### Virtualized SIMD vector programming model

Because a warp executes one common instruction at a time, all threads in a warp are implicitly synchronized after executing any instruction. This execution manner is very similar to the characteristic of SIMD vector organizations that a single instruction controls multiple processing elements. Therefore, it is viable to virtualize a warp as an SIMD vector with each thread as a vector element. An alternative virtualization at the warp level is to divide a warp into several thread groups of equal size and then virtualize each thread group as a vector with each thread in the group as an element. However, for the current CUDA-enabled GPU technologies, this warp-level virtualization limits the virtualized vector length to 32. To support longer vector lengths, vectors can be virtualized at the thread-block level, where a thread block is considered as a large vector with each thread in the thread block as an element. In this case, the intrinsic function __*syncthreads*() has to be used to explicitly synchronize all threads at specific synchronization points in the kernel to keep the correctness of the virtualized vector computations.

In this paper, we refer to the virtualized vector as *virtualized SIMD vector *and its corresponding programming model as *virtualized SIMD model *to differentiate from the real SIMD vector organizations. Since this virtualization is based on the SIMT model, the virtualized SIMD model shares all the features of the SIMT model with an additional ability to conduct vector computations. We define *VL *to denote the length of a virtualized vector, i.e. the number of data lanes of the vector. For the convenience of discussion, we assume that the first element (indexed by 0) is on the rightmost and the last element (indexed by *VL *- 1) on the leftmost of the vector. Each thread comprising a virtualized vector is assigned a vector ID *vtid *that is equal to the position index of its corresponding vector element in the vector of length *VL*, where 0 ≤ *vtid *<*VL*. In this paper, we use warp-level virtualization to implement vectorized SW algorithms.

## Methods

### Query profile

To calculate *H*(*i*, *j*), the substitution score *sbt*(*S*_1_[*i*], *S*_2_[*j*]), from the substitution matrix, is added to *H*(*i*-1, *j*-1). Due to the huge number of iterations in the SW algorithm calculation, reducing the number of instructions needed to perform one cell calculation has a significant impact on the execution time. In this regard, Rognes et al. [10] and Farrar [11] suggested the use of a query profile parallel to the query sequence for each possible residue. A query profile is pre-calculated just once before database searches, and can be calculated in two layouts: a sequential layout [10] and a striped layout [11].

Given a query sequence *Q *of length *l *defined over an alphabet Σ, a query profile is defined as a numerical string set *P *= {*P*_*r *_| *r *∈ Σ}, where *P*_*r *_is a numeric string consisting of substitution scores that are required to compute a complete column (or row) of the alignment matrix, and the values of *P*_*r *_depend on whether it uses a sequential or a striped layout. For a sequential query profile (see Additional file 2), *P*_*r*_(*i*), the *i*-th value of *P*_*r*_, is defined as(2)

Even though a sequential query profile is initially designed for SIMD vector computation of the SW algorithm, it is also suitable for scalar computation of the algorithm. For SIMD vector computation, it generally aligns *l *according to vector length *VL *for performance consideration and pads *Q *with dummy residues that have a substitution score of zero between itself and any residue.

A striped query profile (see Additional file 3) is designed for SIMD vector computation. To construct a striped query profile, given a vector length *VL*, the query sequence *Q *is divided into a set of equal length query segments *QSEG *= {*QSEG*_1_, *QSEG*_2_, ..., *QSEG*_*VL*_} of *VL *elements. The length *T *of each query segment is equal to (*l *+ *VL *- 1)/*VL*. If *l *is not a multiple of *VL*, *Q *is first padded with dummy residues. For simplicity, we assume *l *is a multiple of *VL*. Correspondingly, each numerical string *P*_*r *_of a striped query profile can be considered as a set of non-overlapping, consecutive *VL*-length vector segments *VSEG *= {*VSEG*_1_, *VSEG*_2_, ..., *VSEG*_*T*_} of *T *elements, where the *i*-th element of *VSEG*_*j *_maps the *j*-th element of *QSEG*_*i*_. Hence, *P*_*r*_(*i*) of a striped query profile is defined as(3)

### Optimized SIMT Smith-Waterman algorithm using CUDA

The SIMT SW algorithm used by CUDASW++ 1.0 is designed based on the SIMT abstraction of CUDA-enabled GPUs, which enables thread-level parallelism for independent scalar threads as well as data parallelism for coordinated threads. It uses two stages to complete the database searches: the first stage uses inter-task parallelization using thread-level parallelism, and the second stage uses intra-task parallelization using data parallelism. Since the first stage dominates the total runtime when searching large database, the optimizations of CUDASW++ 2.0 are focused on this stage. The performance of CUDASW++ 2.0 is significantly improved due to the following optimizations: introducing a sequential query profile and using a packed data format.

**Figure 1:**
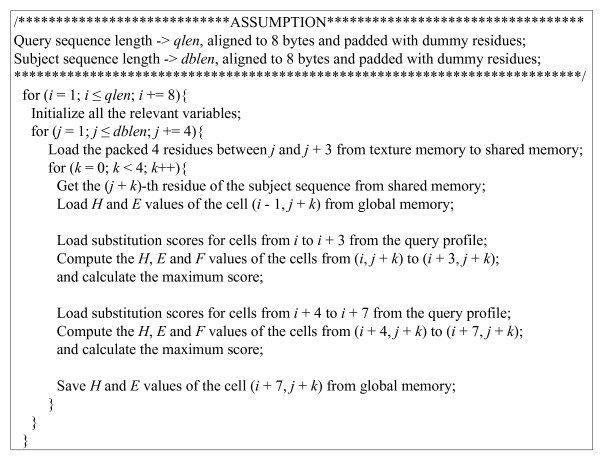
**Pseudocode of the CUDA kernel for the optimized SIMT algorithm**.

A sequential query profile stored in texture memory is used to replace random access to the substitution matrix in shared memory. Inspired by the fact that texture instructions output filtered samples, typically a four-component (RGBA) color [19], the sequential query profile is re-organized using a packed data format, where each numerical string *P*_*r *_is packed and represented using the *char*4 vector data type, instead of the *char *scalar data type. In this way, four substitution scores are realized using only one texture fetch, thus significantly improving texture memory throughput. Like the query profile, each subject sequence is also re-organized using a packed data format, where four successive residues of each subject sequence are packed together and represented using the *uchar4 *vector data type. In this case, when using the cell block division method, the four residues loaded by one texture fetch are further stored in shared memory for the use of the inner loop (see the pseudocode of the CUDA kernel shown in Figure 1). In addition, some redundant operations are removed to improve instruction pipeline throughput.

### Basic vectorized Smith-Waterman algorithm using CUDA

The basic vectorized SW algorithm is designed by directly mapping the striped SW algorithm [11] onto CUDA-enabled GPUs using CUDA, based on the virtualized SIMD vector programming model. For the computation of each column of the alignment matrix, the striped SW algorithm consists of two loops: an inner loop calculating local alignment scores postulating that *F *values do not contribute to the corresponding *H *values, and a lazy-F loop correcting any errors introduced from the calculations of the inner loop. The basic vectorized algorithm uses a striped query profile. In the alignment matrix, for a specific column, the inner loop is completed in *T *iterations by moving SIMD vectors sequentially through all vector segments of *P*_*r *_corresponding to this column. For the convenience of discussion, define *vecH*(*i*, *j*), *vecE*(*i*, *j*) and *vecF *to process the *H*, *E *and *F *values of the cells corresponding to *VSEG*_*i *_of *P*_*r*_, where 1 ≤ *i *≤ *T*, for the *j*-th column of the alignment matrix. Using virtualized SIMD vectors, several technical issues have to be addressed for this CUDA algorithm, including saturation algorithmic operations, shift operations and predicate operations on virtualized vectors.

Saturation additions and subtractions are required to calculate alignment scores. Since CUDA-enabled graphics hardware lacks support for these operations, maximum and minimum operations are used to artificially implement them. The integer functions *max(x, y) *and *min(x, y)*, in the CUDA runtime library, are used to avoid divergence. Shift operations on vectors are required both for the inner and lazy-F loops. We implement these operations using shared memory, where all threads comprising a virtualized vector writes their original values to a share memory buffer and then reads their resulting values from the buffer as per the number of shift elements. Additional file 4 gives the CUDA pseudocode for shifting a virtualized vector by *n *elements to the left. As can be seen from the pseudocode, one shift operation is time-consuming as compared with vector register operations in a real SIMD vector architectures, even though access to shared memory without bank conflicts has a much lower latency than device memory [20].

The lazy-F loop requires predicate operations on virtualized vectors when determining whether to continue or exit the loop by checking *vecF *against the values of *vecH*(*i*, *j*). An approach is to use shared memory to simulate these operations. Although this approach is effective, it is inefficient due to the overhead incurred by the accesses to shared memory. Fortunately, CUDA-enabled GPU devices with compute capability 1.2 and higher provide the support for two warp vote functions __*all*(*int*) and *__any*(*int*), providing an indispensible capability to perform fast predicate operations across all threads within a warp. We use the __*all*(*int*) warp vote function to implement the predicate operations on virtualized vectors for the lazy-F loop.

The striped query profile is stored in texture memory to exploit the texture cache. Subject sequences and the query profile are stored using the scalar data type in an unpacked fashion because the inner loop is a *for *loop without manually unrolling. The intermediate element values of *vecH*(*i*, *j*) and *vecE*(*i, j*) are stored in global memory, with *vecF *stored in registers, to support long query sequences. To improve global memory access efficiency, we use the unsigned half-word data type to store the *H *and *E *values in global memory.

### Partitioned vectorized Smith-Waterman algorithm using CUDA

To gain higher performance, we have investigated a partitioned vectorized SW algorithm using CUDA. This algorithm first divides a query sequence into a series of non-overlapping, consecutive small partitions as per a specified partition length (*PL*), and then aligns the query sequence to a subject sequence partition by partition. For the partitioned vectorized algorithm, *PL *must be a multiple of *VL*. The alignment between one partition of the query sequence and the subject sequence is performed using the basic vectorized algorithm. In this case, because *PL *is usually set to be relatively smaller, shared memory or registers can be used to store the alignment scores.

**Figure 2:**
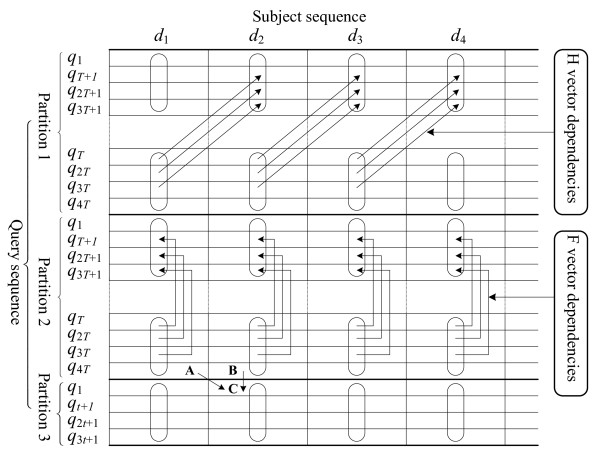
**Alignment matrix of the partitioned vectorized algorithm and data dependencies for H and F vectors**.

In this algorithm, it considers each partition as a new query sequence and constructs a striped query profile for each partition. However, this partitioned vectorized algorithm makes the alignment of one partition depend on the alignment scores of the previous partition in the alignment matrix (see Figure 2). More specifically, the computation of the *H *and *F *values of the first row of one partition depends on the *H *and *F *values of the last row of the previous partition (note that the positions of the first and the last rows are kept unchanged for a striped query profile regardless of the specific values of *PL *and *VL*). In this case, after having completed the computation of one column of a partition, the *H *and *F *values of the last cell in this column have to be saved for the future use of the next partition. For performance consideration, instead of directly storing *F *value of the last cell of the partition, we store the *F *value of the first cell of the next partition, calculated from the *H *and *F *values of the last cell in the current partition. However, there is a problem with the striped algorithm in that for a specific column, after exiting from the lazy-F loop, it makes sure that the *H *and *E *values of all cells are correct, but provides no guarantee that *vecF *stores the correct *F *value of the last cell. This is because the lazy-F loop is likely to quit, with high probability, with no need to re-calculate all the cell values. Since the *H *values of all cells in the last row of the previous partition are always correct, the correctness of our partitioned vectorized algorithm will be proved if we could prove that the correctness of the *F *values of all cells in the last row of the previous partition does not affect the correct computation of *F *values of all cells in the first row of the current partition. In the following, we will prove that the *F *values of all cells in the first row of the current partition can always be correctly calculated.

**Theorem 1**. For the partitioned vectorized SW algorithm, the *F *values of all cells in the first row of the current partition are correctly computed regardless of the correctness of the *F *values of all cells in the last row of its previous partition.

**Proof**. Taking cells *B *and *C *in Figure 2 as an example, define *C*_*F *_to denote the *F *value of *C*, *B*_*H *_to denote the *H *value of *B*, and *B*_*F *_to denote the *F *value of *B*, where *C*_*F *_= *max*(*B*_*H *_- *ρ *- *σ*, *B*_*F *_- *σ*) according to equation (1). For the striped SW algorithm, the correctness of the *F *value of the last cell for a specific column *j *in the alignment matrix depends on two possible conditions.

*Case *1: the lazy-F loop does not stop until the *F *values of all cells in column *j *have been corrected because the *F *value of each cell contributes to its *H *value. In this case, due to the recalculation of all cells, *vecF *stores the correct *F *value of the last cell. Since both *B*_*H *_and *B*_*F *_are correct, *C*_*F *_is definitely correctly calculated.

*Case *2: the lazy-F loop stops after some iterations with no need to recalculate all cells. This means that the *F *values of the remaining cells will not contribute to their corresponding *H *values, but might not be equal to their correct *F *values directly calculated using equation (1). In this case, because *B*_*F *_- *σ *≤ *B*_*H *_- *ρ *- *σ*, *C*_*F *_is equal to *B*_*H *_- *ρ *- *σ *so that *C*_*F *_has no relationship to *B*_*F*_.

From the above discussion, a conclusion can be drawn that *C*_*F *_can always be correctly calculated regardless of whether *B*_*F *_is correct or not. Therefore, the theorem is proven.

**Figure 3:**
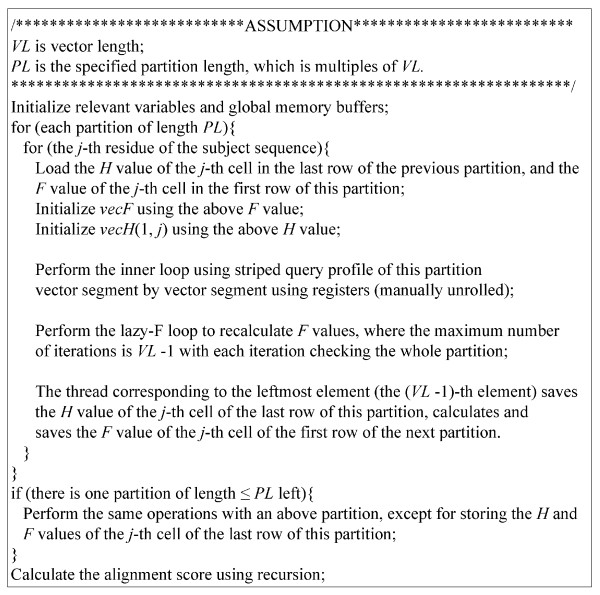
**Pseudocode of CUDA kernel of the partitioned vectorized Smith-Waterman algorithm**.

The partitioned vectorized algorithm stores the values of *vecH *and *vecE *of one column for a partition in registers to achieve peak performance. Using registers, the inner loop and the lazy-F loops are manually unrolled according to the number of vector segments. Since the computation of the inner loop is fully unrolled, the access to the striped query profile can be optimized using the packed data format. Like the optimized SIMT algorithm, each numerical string *P*_*r *_of this query profile is packed and represented using the *char*4 vector data type according to the access order of threads comprising a virtualized vector. However, the subject sequences are not packed because we failed to find performance improvement using the packed data format. Figure 3 shows the pseudocode of the CUDA kernel of the partitioned vectorized algorithm.

## Results and discussion

We use GCUPS [17] to measure the performance of our algorithms. In this paper, the execution time *t *includes the transfer time of the query sequences from host to GPU, the calculation time of the SW algorithm, and the time taken to transfer-back the scores. In addition, when running on multiple GPUs, *t *also includes the transfer time of database sequences from host memory to GPU, and time required for creating and destroying the host threads.

The performance of CUDASW++ 2.0 is benchmarked and analyzed by searching for 20 sequences of length from 144 to 5,478 against Swiss-Prot release 56.6 (released on Dec. 16, 2008, comprising 146,166,984 amino acids in 405,506 sequences and having the longest sequence of 35,213 amino acids). The tests on a single GPU are carried out on a GeForce GTX 280 (GTX 280) graphics card, with 30 SMs comprising 240 SPs and 1 GB RAM, installed in a PC with an AMD Opteron 248 2.2 GHz processor running the Linux OS. The multiple GPU tests are carried out on a GeForce GTX 295 (GTX 295) graphics card with two G200 GPU-chips on a single card, which consists of 480 SPs (240 SPs per GPU) and 1.8 GB RAM, installed in a PC with an Intel *i*7 quad-core 2.67 GHz processor running the Linux OS. This graphics card has a slightly lower clock frequencies compared to GTX 280.

The performance of the optimized SIMT algorithm has no relationship with the substitution matrix and gap penalties used, whereas the two vectorized algorithms are sensitive to them. Generally, for a specific substitution matrix, the higher the gap open and gap extension penalties, the higher the performance. This is because fewer iterations are needed to recalculate *F *in the lazy-F loop. Since the BLOSUM family of substitution matrices, particularly BLOSUM62, is the *de facto *standard in protein database searches and sequence alignments, if not specified, all the tests in this paper use BLOSUM62 as the substitution matrix by default. The optimized SIMT algorithm exploits a gap penalty of 10-2 k, and the two vectorized algorithms use several different gap penalties to check the runtime characteristics. For the optimized SIMT algorithm, maximal performance is achieved for a thread block size of 256 threads and a grid size equal to 4× the number of SMs; for the basic vectorized algorithm, maximal performance is achieved using *VL *equal to warp size (i.e. 32) for a thread block of 256 threads and a grid size equal to 64× the number of SMs; and for the partitioned vectorized algorithm, maximal performance is achieved using *PL *equal to 256 and *VL *equal to half-warp size (i.e. 16) for a thread block of 192 threads and a grid size equal to 128× the number of SMs. The basic vectorized algorithm produces much lower performance than the partitioned vectorized algorithm for several different gap penalties. Additional file 5 shows the performance percentage ratio of the basic vectorized algorithm to the partitioned vectorized one for different gap penalties on a single GPU. Hence, the basic vectorized algorithm is excluded from the release of CUDASW++ 2.0.

The optimized SIMT algorithm achieves an average performance of 16.5 (27.2) GCUPS with a highest of 16.9 (28.8) GCUPS on GTX 280 (GTX 295). The partitioned vectorized algorithm achieved an average performance of 15.3 (22.9) GCUPS with a highest of 16.3 (27.0) GCUPS using a gap penalty of 10-2 k; an average performance of 16.3 (24.8) GCUPS with a highest of 17.6 (29.6) GCUPS using a gap penalty of 20-2 k; and an average performance of 16.8 (26.2) GCUPS with a highest of 17.8 (29.7) GCUPS using a gap penalty of 40-3 k on GTX 280 (GTX 295). The runtime (in seconds) and GCUPS of the optimized SIMT and partitioned vectorized algorithms on GTX 280 and GTX 295 is shown in Tables 1 and 2, respectively. From the tables, it can be seen that the optimized SIMT algorithm produces reasonably stable performance, while the performance of the partitioned vectorized algorithm shows some small fluctuations around the average performance, increasing with the increase of the gap open and gap extension penalties. On GTX 280, the optimized SIMT algorithm slightly outperforms the partitioned vectorized algorithm using a gap penalty of 10-2 k, has nearly the same performance with the latter using a gap penalty of 20-2 k, but is slightly outperformed when using a gap penalty of 40-3 k. On GTX 295, the optimized SIMT algorithm has a slightly higher average performance than the partitioned vectorized algorithm using any of the three gap penalties, but has a lower highest performance when using gap penalties of 20-2 k and 40-3 k. This indicates that these two algorithms have remarkably similar performance characteristics.

**Table 1:** Performance evaluation of the optimized SIMT and partitioned vectorized algorithms on GTX 280

Query Sequences		Partitioned						SIMT	
		
		10-2 k		20-2 k		40-3 k		10-2 k	
**Query**	**Length**	**Time**	**GCUPS**	**Time**	**GCUPS**	**Time**	**GCUPS**	**Time**	**GCUPS**

P02232	144	1.58	13.3	1.41	14.9	1.40	15.0	1.38	15.2

P05013	189	1.80	15.4	1.66	16.7	1.65	16.8	1.75	15.8

P14942	222	2.01	16.1	1.84	17.6	1.82	17.8	2.00	16.2

P07327	375	3.97	13.8	3.64	15.1	3.51	15.6	3.35	16.4

P01008	464	4.57	14.8	4.20	16.1	4.03	16.8	4.05	16.7

P03435	567	5.87	14.1	5.38	15.4	5.28	15.7	4.94	16.4

P42357	657	6.64	14.5	6.16	15.6	5.97	16.1	5.00	16.6

P21177	729	6.92	15.4	6.40	16.6	6.24	17.1	5.77	16.6

Q38941	850	7.98	15.6	7.37	16.9	7.35	16.9	6.35	16.8

P27895	1000	10.27	14.2	9.29	15.7	8.74	16.7	7.44	16.7

P07756	1500	15.07	14.5	14.08	15.6	13.43	16.3	8.64	16.9

P04775	2005	19.30	15.2	18.05	16.2	17.36	16.9	13.04	16.8

P19096	2504	22.89	16.0	21.49	17.0	21.19	17.3	17.50	16.7

P28167	3005	28.54	15.4	26.08	16.8	25.53	17.2	21.89	16.7

P0C6B8	3564	32.44	16.1	30.56	17.0	29.60	17.6	26.41	16.6

P20930	4061	40.47	14.7	36.07	16.5	34.31	17.3	31.35	16.6

P08519	4548	42.41	15.7	39.89	16.7	38.86	17.1	35.84	16.6

Q7TMA5	4743	42.44	16.3	39.36	17.6	39.30	17.6	40.18	16.5

P33450	5147	50.91	14.8	47.74	15.8	44.20	17.0	41.92	16.5

Q9UKN1	5478	55.46	14.4	49.49	16.2	46.66	17.2	45.62	16.5

**Table 2:** Performance evaluation of the optimized SIMT and partitioned vectorized algorithms on GTX 295

Query Sequences		Partitioned						SIMT	
		
		10-2 k		20-2 k		40-3 k		10-2 k	
**Query**	**Length**	**Time**	**GCUPS**	**Time**	**GCUPS**	**Time**	**GCUPS**	**Time**	**GCUPS**

P02232	144	1.19	17.7	1.13	18.7	1.09	19.4	1.02	20.7

P05013	189	1.34	20.7	1.30	21.4	1.26	22.1	1.25	22.3

P14942	222	1.49	22.0	1.41	23.1	1.38	23.7	1.37	23.8

P07327	375	2.77	19.9	2.58	21.4	2.42	22.8	2.15	25.7

P01008	464	3.04	22.4	2.82	24.2	2.66	25.6	2.54	26.8

P03435	567	3.93	21.2	3.61	23.1	3.49	23.9	3.11	26.8

P42357	657	4.29	22.5	4.02	24.0	3.87	25.0	3.56	27.1

P21177	729	4.53	23.7	4.22	25.4	4.04	26.5	3.90	27.5

Q38941	850	5.03	24.9	4.66	26.8	4.63	27.0	4.53	27.6

P27895	1000	6.58	22.3	5.87	25.1	5.38	27.3	5.21	28.2

P07756	1500	9.86	22.4	9.19	24.0	8.58	25.7	7.72	28.6

P04775	2005	12.26	24.1	11.32	26.0	10.79	27.3	10.26	28.7

P19096	2504	14.32	25.7	13.34	27.6	12.99	28.4	12.79	28.8

P28167	3005	18.31	24.1	16.46	26.9	15.56	28.4	15.33	28.8

P0C6B8	3564	21.09	24.9	19.34	27.1	17.99	29.1	18.20	28.8

P20930	4061	26.75	22.3	23.35	25.6	20.76	28.8	20.77	28.8

P08519	4548	27.36	24.4	25.11	26.6	23.92	28.0	23.24	28.8

Q7TMA5	4743	25.86	27.0	23.57	29.6	23.51	29.7	24.24	28.8

P33450	5147	32.69	23.2	30.57	24.8	27.37	27.7	26.33	28.7

Q9UKN1	5478	36.61	22.0	32.40	24.9	28.88	27.9	28.05	28.7

**Figure 4:**
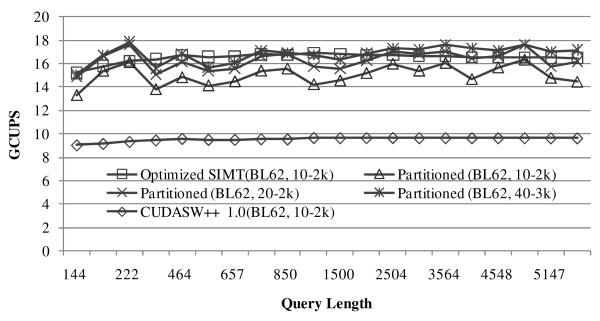
**Performance comparison between CUDASW++ 1.0 and CUDASW++ 2.0 on GTX 280**.

**Figure 5:**
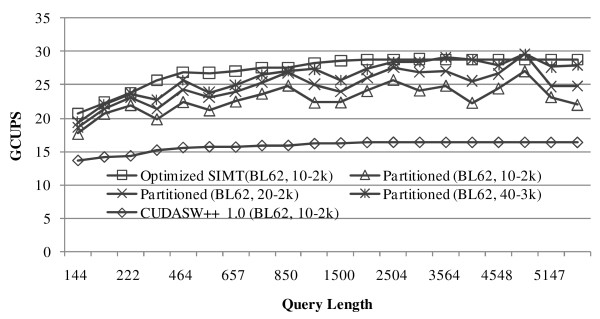
**Performance comparison between CUDASW++ 1.0 and CUDASW++ 2.0 on GTX 295**.

We next compare CUDASW++ 2.0 to CUDASW++ 1.0. CUDASW++ 1.0 is re-benchmarked on the same platforms as used for CUDASW++ 2.0. Since the performance of CUDASW++ 1.0 is not affected by the choice of substitution matrix and gap penalty, we use BLOSUM62 with a gap penalty of 10-2 k for all tests. Figure 4 and Figure 5 show the performance comparison between CUDASW++ 1.0 and CUDASW++ 2.0 on GTX 280 and GTX 295, respectively. On average, compared to CUDASW++ 1.0, the optimized SIMT algorithm runs about 1.74 (1.72) times faster on GTX 280 (GTX 295); the partitioned vectorized algorithm runs about 1.58 (1.45) times faster using a gap penalty of 10-2 k, about 1.72 (1.57) times faster using a gap penalty of 20-2 k, and about 1.77 (1.66) times faster using a gap penalty of 40-3 k on GTX 280 (GTX 295). Hence, CUDASW++ 2.0 obtains significant performance improvement over CUDASW++ 1.0 using either the optimized SIMT or the partitioned vectorized algorithms running on the same platforms.

We decided not to include comparisons with the following publicly available SW implementations: SWPS3, SW-CUDA, and CBESW, as CUDASW++ 1.0 significantly outperforms them. Now, we compare the performance between CUDASW++ 2.0 and NCBI-BLAST (version 2.2.22+). NCBI-BLAST is re-benchmarked on the above PC with an Intel *i*7 quad-core processor. The substitution matrices BLOSUM62 with a gap penalty of 10-2 k and BLOSUM50 with a gap penalty of 10-3 k are used for the tests. All the other parameters are used by default. To demonstrate the power of CUDASW++ 2.0 for long query sequences, we build a subset of Swiss-Prot release 56.6 database, which contains all the sequences of lengths ≥ 2000 in the database. This subset comprises 5,670,072 amino acids in 1,875 sequences. Performance is compared by searching all sequences in this subset against the Swiss-Prot database. As mentioned above that the performance of CUDASW++ 1.0 and the optimized SIMT algorithm of CUDASW++ 2.0 has no relationship with the substitution matrix and gap penalties used, we just test them using BLOSUM62 with a gap penalty of 10-2 k. Table 3 shows the runtime (in hours) and average GCUPS of CUDASW++ 1.0, CUDASW++ 2.0 and NCBI-BLAST, where CUDASW++ 1.0 and 2.0 are benchmarked on GTX 295. From the table, CUDASW++ 2.0 using the optimized SIMT algorithm produces the best performance, taking 8.00 hours to complete the searching and achieving an average of 28.8 GCUPS. NCBI-BLAST using BLOSUM50 and a gap penalty of 10-3 k produces the worst performance, taking up to 51.45 hours and achieving only an average of 4.5 GCUPS. Even though the partitioned vectorized algorithm gives lower performance than the optimized SIMT algorithm due to the use of smaller gap penalties, it still significantly outperforms CUDASW++ 1.0 and NCBI-BLAST that uses BLOSUM50 and a gap penalty of 10-3 k. Hence, the overall performance of CUDASW++ 2.0 is significantly better as compared with CUDASW++ 1.0 and NCBI-BLAST.

**Table 3:** Performance comparison between CUDASW++ 1.0, CUDASW++ 2.0 and NCBI-BLAST

Software	Performance	
	
	Time(h)	GCUPS
Optimized SIMT (BL62, 10-2 k)	8.00	28.8

Partitioned (BL62, 10-2 k)	11.15	20.7

Partitioned (BL50, 10-3 k)	11.71	19.7

NCBI-BLAST(BL62, 10-2 k)	9.56	24.1

NCBI-BLAST(BL50, 10-3 k)	51.45	4.5

CUDASW++ 1.0 (BL62, 10-2 k)	14.12	16.3

## Conclusions

In this paper, we have presented our new contributions to SW database searches using CUDA, through the latest release of the CUDASW++ 2.0 software targeted for CUDA-enabled GPUs with compute capability 1.2 and higher. An optimized SIMT SW algorithm is suggested to further optimize the performance of CUDASW++ 1.0 based on the SIMT abstraction of CUDA-enabled GPUs. For the first time we have investigated a partitioned vectorized SW algorithm using CUDA based on the virtualized SIMD abstraction of CUDA-enabled GPUs. This virtualized SIMD vector programming model provides guidance for designing other bioinformatics algorithms, such as pairwise distance computation in ClustalW [21,22], using SIMD vectorization for CUDA-enabled GPUs. The optimized SIMT and the partitioned vectorized algorithms have remarkably similar performance characteristics when benchmarked by searching the Swiss-Prot release 56.6 database with query sequences of length varying from 144 to 5,478. The optimized SIMT algorithm produces reasonably stable performance, while the partitioned vectorized algorithm has some small fluctuations around the average performance for a specific gap penalty, increasing with the increase of the gap open and gap extension penalties. CUDASW++ 2.0 provides direct support for multiple GPU devices installed in a single host. It obtains significant performance improvement over CUDASW++ 1.0 using either the optimized SIMT algorithm or the partitioned vectorized algorithm on the same platform, achieving a highest performance of up to 17 (30) GCUPS on GTX 280 (GTX 295).

Even though the optimal alignment scores of the SW algorithm can be used to detect related sequences, the scores are biased by sequence length and composition. The Z-value [23-25] has been proposed to estimate the statistical significance of these scores. However, the computation of Z-value requires the calculating of a large set of pairwise alignments between random permutations of the sequences compared, which is highly time-consuming. The acceleration of Z-value computation with CUDA is therefore part of our future work.

## Availability and requirements

• **Project name**: CUDASW++

• **Project home page**: http://cudasw.sourceforge.net/

• **Operating System**: Linux

• **Programming language**: CUDA and C++

• **Other requirements**: CUDA SDK and Toolkits 2.0 or higher; CUDA-enabled GPUs with compute capability 1.2 and higher

• **License**: none

## List of abbreviations

CPU: Central Processing Unit; CUDA: Compute Unified Device Architecture; Cell/BE: Cell Broadband Engine Architecture; FPGA: Field-Programmable Gate Array; GCPUS: Billion Cell Updates per Second; GPU: Graphics Processing Unit; GTX 280: NVIDIA GeForce GTX 280; GTX 295: NVIDIA GeForce GTX 295; OpenGL: Open Graphics Library; OS: Operating System; PBSM: Per-block Shared Memory; PC: Personal Computer; RAM: Random Access Memory; SIMD: Single Instruction Multiple Data; SIMT: Single-instruction, Multiple-thread; SM: Streaming Multiprocessor; SP: Scalar Processor; SSE2: Streaming SIMD Extensions 2; SW: Smith-Waterman.

## Competing interests

The authors declare that they have no competing interests.

## Authors' contributions

YL conceptualized the study, carried out the design and implementation of the algorithm, performed benchmark tests, analyzed the results and drafted the manuscript; BS conceptualized the study, participated in the algorithm optimization and analysis of the results and contributed to the revising of the manuscript; DLM conceptualized the study and contributed to the revising of the manuscript. All authors read and approved the final manuscript.

## Supplementary Material

Additional file 1 **Data dependencies in the Smith-Waterman alignment matrix**. This figure demonstrates the data dependencies in the alignment matrix for the Smith-Waterman algorithm. Click here for file

Additional file 2 **An example query profile using sequential layout**. This figure demonstrates an example query profile using sequential layout. Click here for file

Additional file 3 **An example query profile using striped layout**. This figure demonstrates an example query profile using striped layout, where *VL *= 4 and *T *= 4. Click here for file

Additional file 4 **CUDA pseudocode of shifting a virtualized vector by *n *elements to the left**. This figure demonstrates an example CUDA pseudocode of shifting a virtualized vector by *n *elements to the left. Click here for file

Additional file 5 **Performance percentage ratio of the basic vectorized algorithm to the partitioned vectorized one**. This figure demonstrates the performance percentage ratio of the basic vectorized algorithm to the partitioned vectorized one on a single GPU. Click here for file
