# Supplementary material for: CUDASW++2.0: enhanced Smith-Waterman protein database search on CUDA-enabled GPUs based on SIMT and virtualized SIMD abstractions
Source: BMC Res Notes. 2010 Apr 6;3:93. doi: 10.1186/1756-0500-3-93 (PMC2907862; doi:10.1186/1756-0500-3-93)
Supplement: Supplementary file 1 [file 1756-0500-3-93-S1.DOC]

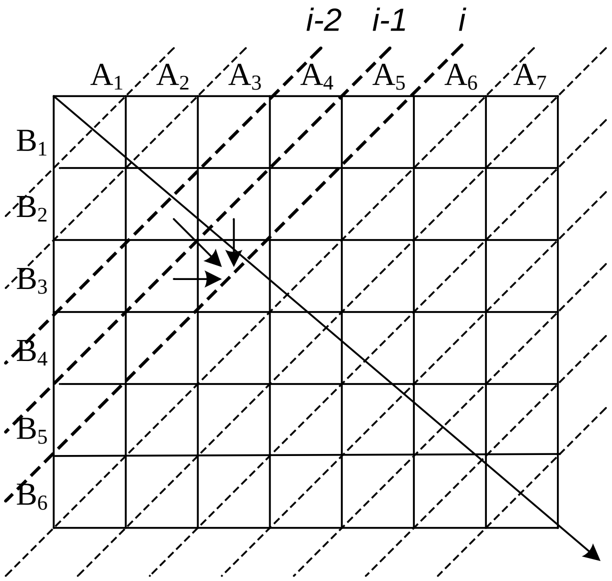

i-2
i-1
i
A1
A2
A3
A4
A5
A6
A7
B1
B2
B3
B4
B5
B6
