# Supplementary material for: CUDASW++2.0: enhanced Smith-Waterman protein database search on CUDA-enabled GPUs based on SIMT and virtualized SIMD abstractions
Source: BMC Res Notes. 2010 Apr 6;3:93. doi: 10.1186/1756-0500-3-93 (PMC2907862; doi:10.1186/1756-0500-3-93)
Supplement: Supplementary file 2 [file 1756-0500-3-93-S2.DOC]

Query Sequence

Residue Σ	q_1	q_2	q_3	q_4	q_5	q_6	q_7	q_8	q_9	q_{10}	q_{11}	q_{12}	q_{13}	q_{14}	q_{15}	q_{16}	
A	-1	0	-2	0	-1	-1	-1	-1	5	-1	0	-1	1	1	1	-1	
B	-2	-3	-2	-1	-3	-3	-3	-3	-1	-2	-1	-2	-1	-1	-1	-3	P_B
⋮			⋮					⋮						⋮			
Y	0	-3	3	-1	-2	-1	-1	-2	-2	0	-1	-2	-2	-2	-2	-1	P_Y
Z	-1	-2	-3	-1	4	1	4	4	-1	-2	-3	0	0	0	0	4	
