# Supplementary material for: CUDASW++2.0: enhanced Smith-Waterman protein database search on CUDA-enabled GPUs based on SIMT and virtualized SIMD abstractions
Source: BMC Res Notes. 2010 Apr 6;3:93. doi: 10.1186/1756-0500-3-93 (PMC2907862; doi:10.1186/1756-0500-3-93)
Supplement: Supplementary file 3 [file 1756-0500-3-93-S3.DOC]

Query Sequence

Residues in Σ	$VSEG_1$				$VSEG_2$				$VSEG_3$				$VSEG_4$				
	q_1	q_{2T+1}	q_{3T+1}	q_{4T+1}	q_2	q_{2T+2}	q_{3T+2}	q_{4T+2}	q_3	q_{2T+3}	q_{3T+3}	q_{4T+3}	q_4	q_{2T+4}	q_{3T+4}	q_{4T+4}	
A	-1	-1	5	1	0	-1	-1	1	-2	-1	0	1	0	-1	-1	-1	
B	-2	-3	-1	-1	-3	-3	-2	-1	-2	-3	-1	-1	-1	-3	-2	-3	P_B
⋮		⋮						⋮						⋮			
Y	0	-2	-2	-2	-3	-1	0	-2	3	-1	-1	-2	-1	-2	-2	-1	P_Y
Z	-1	4	-1	0	-2	1	-2	0	-3	4	-3	0	-1	4	0	4	
