# Supplementary material for: CUDASW++2.0: enhanced Smith-Waterman protein database search on CUDA-enabled GPUs based on SIMT and virtualized SIMD abstractions
Source: BMC Res Notes. 2010 Apr 6;3:93. doi: 10.1186/1756-0500-3-93 (PMC2907862; doi:10.1186/1756-0500-3-93)
Supplement: Supplementary file 4 [file 1756-0500-3-93-S4.DOC]

/*vecShift is a pointer to shared memory, and regElement is an element value of the virtualized vector corresponding to thread *vtid* comprising the vector, where $0 \le vtid < VL$*/

```
vecShift[vtid] = regElement;
regElement = 0;
if (vtid >= n){
    regElement = vecShift[vtid – n];
}
```
