# Supplementary material for: CUDASW++2.0: enhanced Smith-Waterman protein database search on CUDA-enabled GPUs based on SIMT and virtualized SIMD abstractions
Source: BMC Res Notes. 2010 Apr 6;3:93. doi: 10.1186/1756-0500-3-93 (PMC2907862; doi:10.1186/1756-0500-3-93)
Supplement: Supplementary file 5 [file 1756-0500-3-93-S5.DOC]

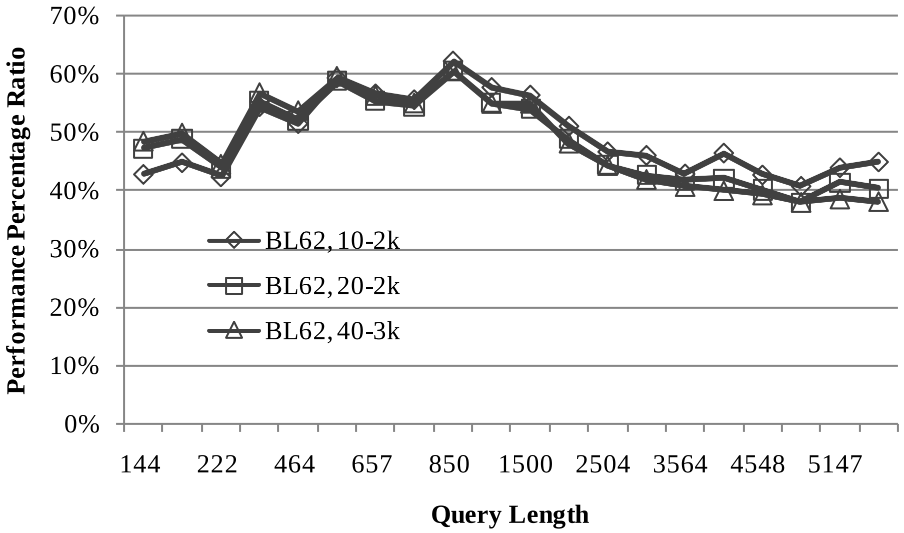

Performance Percentage Ratio
70%
60%
50%
40%
30%
20%
10%
0%
144
222
464
657
850
1500
2504
3564
4548
5147
Query Length
BL62, 10-2k
BL62, 20-2k
BL62, 40-3k
